# Proposal of an Algorithm to Early Detect Attenuated Type I Mucopolysaccharidosis (MPS Ia) among Children with Growth Abnormalities

**DOI:** 10.3390/medicina58010097

**Published:** 2022-01-08

**Authors:** Federico Baronio, Stefano Zucchini, Francesco Zulian, Mariacarolina Salerno, Rossella Parini, Alessandro Cattoni, Federica Deodato, Alberto Gaeta, Carla Bizzarri, Serena Gasperini, Andrea Pession

**Affiliations:** 1 Department of Medical and Surgical Sciences, University of Bologna, 40138 Bologna, Italy; stefano.zucchini@aosp.bo.it (S.Z.); andrea.pession@unibo.it (A.P.); 2 Rheumatology Unit, Department of Woman’s and Child’s Health, University of Padua, 35128 Padua, Italy; francesco.zulian@unipd.it; 3 Pediatric Endocrine Unit, Department of Translational Medical Sciences, University of Naples Federico II, 80131 Naples, Italy; salerno@unina.it; 4 Department of Pediatrics, Milano-Bicocca University, Fondazione MBBM, San Gerardo Hospital, 20900 Monza, Italy; rossella.parini@unimib.it (R.P.); alessandro.cattoni@unimib.it (A.C.); serena.gasperini69@gmail.com (S.G.); 5 Division of Metabolic Disease, Bambino Gesù Children’s Hospital IRCSS, 00165 Rome, Italy; federica.deodato@opbg.net; 6 Radiology Unit, Pediatric Hospital Giovanni XXIII, 70123 Bari, Italy; al.gaeta@libero.it; 7 Unit of Endocrinology, Bambino Gesù Children’s Hospital, 00165 Rome, Italy; carla.bizzarri@opbg.net

**Keywords:** mucopolysaccharidosis, short stature, joint contractures, dysostosis multiplex, diagnostic algorithm

## Abstract

*Background and Objectives:* Diagnostic delay is common in attenuated Mucopolysaccharidosis Type I (MPS Ia) due to the rarity of the disease and the variability of clinical presentation. Short stature and impaired growth velocity are frequent findings in MPS Ia, but they rarely raise suspicion as paediatric endocrinologists are generally poorly trained to detect earlier and milder clinical signs of this condition. *Materials and Methods*: Following a consensus-based methodology, a multidisciplinary panel including paediatric endocrinologists, paediatricians with expertise in metabolic disorders, radiologists, and rheumatologists shared their experience on a possible clinical approach to the diagnosis of MPS Ia in children with short stature or stunted growth. *Results*: The result was the formation of an algorithm that illustrates how to raise the suspicion of MPS Ia in a patient older than 5 years with short stature and suggestive clinical signs. *Conclusion*: The proposed algorithm may represent a useful tool to improve the awareness of paediatric endocrinologists and reduce the diagnostic delay for patients with MPS Ia.

## 1. Introduction

Mucopolysaccharidosis type I (MPS I, OMIM 252800) is a progressive and multisystem lysosomal storage disorder due to lysosomal hydrolase α-L-iduronidase deficiency (IDUA, [Genbank NG_008103]).

As a consequence, glycosaminoglycans (GAGs) are stored virtually in all organs, particularly in the connective tissues. This results in a permanent and progressive detrimental effect upon cellular function and affects facial features, body proportions, the functioning of organs and systems and cognitive development [1].

Etiologic treatments are available for MPS I. Enzyme replacement therapy (ERT) reduces urinary GAG secretion, liver volume, and mucosal inflammation, while improving joint mobility, endurance, and respiratory muscle strength [2]. Its main limitation is that it does not cross the blood–brain barrier and has little effect on already established bone deformities and cardiac valves damage. Haematopoietic Stem Cell Transplantation (HSCT) is, at present, the standard-of-care for severe MPS I diagnosed before the age of 2 and with an IQ > 70, as it provides a remarkable improvement in cognitive outcome and growth pattern [3,4].

Pilot studies to detect MPS I with a dried blood spot (DBS) test performed in the first days of life (new-born screening) have been started worldwide [5].

The diagnostic work-up of MPS I includes the quantitative and qualitative dosage of specific urinary GAG (heparan sulphate, dermatan sulphate) and the evaluation of leukocyte enzyme activity, which can be performed also on dried blood spot (DBS) [3].

The clinical phenotype of any patient with MPS I falls within a wide spectrum of severity (“phenotypic continuum”), ranging from Hurler disease, the more severe variant, to Scheie disease, the milder form. All patients with an intermediate phenotype are classified as Hurler–Scheie disease [1].

The most frequent findings in MPS I are reported in Table 1. Short stature and reduced growth velocity are frequently observed [6].

The diagnosis of the attenuated form of MPS I (MPS Ia) may be challenging as the heterogeneity of clinical signs and symptoms are often mild and slowly progressing, and therefore not easily recognizable in early childhood.

This represents the main cause of delay in the diagnosis [7].

Since short stature or impaired growth velocity associated with additional pathognomonic features could raise the clinical suspicion of attenuated MPS Ia [8], diagnostic and treatment delays may be remarkably reduced by improving paediatric endocrinologists’ awareness about milder clinical signs.

We hereby aimed at designing a diagnostic algorithm that may guide paediatric endocrinologists in promptly detecting MPS Ia when assessing a child with short stature or stunted growth.

## 2. Methods

A multidisciplinary panel, including paediatric endocrinologists, paediatricians with expertise in metabolic disorders, radiologists, and rheumatologists came together to share their experience on a possible clinical approach for the diagnosis of MPS Ia in children with short stature or stunted growth. The panel members worked together to outline the general structure of a diagnostic algorithm and its core information flow, including published data, clinical reports, and personal experience. A clinical rationale was identified for each step of the algorithm to increase the adherence to what might happen in real clinical practice. Subsequently, each member suggested improvements to the initial framework, supported by evidence from the literature and by personal experience. Following a consensus-based methodology, the panel of experts reviewed, discussed, and finally implemented the new final version of the algorithm.

## 3. The Rationale for the Algorithm

Children with MPS Ia are commonly diagnosed later than those with Hurler syndrome, due to the milder enzyme deficiency, which leads to slower progression of GAGs storage and the delayed appearance of symptoms. Accordingly, the clinical features of MPS Ia at diagnosis may vary and the concurrent signs and symptoms found mostly depend on the age of the patient. Since MPS Ia patients aged 5–10 years generally show reduced growth velocity [6], this finding should be regarded as the main red flag. Within this age range, joint contractures represent the second most frequently complained symptoms, after cardiac valve abnormalities [9]. More than 80% Hurler–Scheie and Scheie patients experience joint contractures without inflammation, a specific sign that could, per se, raise the suspicion of disease and, if recognized, it could prompt an earlier diagnosis [10].

### 3.1. Growth Impairment in MPS Ia

Short stature is defined as height for chronological age below −2.0 SDS according to the national growth charts.

Children with MPS Ia show growth impairment most frequently during the prepubertal age (5–12 years), whereas growth is generally not impaired in the first two years of life, when it may even be accelerated. According to the data reported by Viskochil et al. [6], the estimated median height for untreated MPS Ia patients falls below the third centile (CDC growth charts) by the age of 9 years. In addition, pubertal spurt tends to be flattened, especially in females.

The pathogenesis of growth impairment in MPS is mostly related to the pathological storage of GAGs in cartilage, bone, and growth plate, resulting in inflammation, apoptosis, and a poorly organized connective tissue matrix [8]. This affects bone growth [3,10,11,12] and leads to disproportionate short stature, with a more remarkable impairment at the trunk level [8,13].

### 3.2. Osteoarticular Involvement in MPS Ia

Osteoarticular abnormalities are commonly observed in patients affected by MPS Ia, in particular joint contractures due to stenosing tenosynovitis (ST), also known as “trigger finger”, skeletal dysostosis, and carpal tunnel syndrome (CTS).

A flexion contracture of the fingers, especially the distal interphalangeal joints, commonly defined as “claw hand”, represents one of the most frequently reported findings among MPS Ia patients.

The limited articular range of motion (ROM) is another hallmark of MPS Ia [14]. It affects hands, elbows, shoulders, hips and knees, with severe and progressive impairment in everyday life tasks.

Upon examination, the physician should assess ROM starting from the fingers and proceeding to elbows, shoulders, hips, and knees. MPS Ia should be suspected in patients with limited joint ROM without local signs of inflammation [10]. Concerning this topic, the pGALS (paediatric gait, arms, legs, and spine) represents a simple musculoskeletal assessment to distinguish abnormal from normal joints in children for the non-specialist in paediatric musculoskeletal medicine. pGALS has been validated in school-aged children and facilitates early recognition of joint-skeletal problems, such as MPS I [15].

### 3.3. Radiological Features of MPS Ia

Defective endochondral and membranous ossification causes dysostosis multiplex, the constellation of clinical and radiographic malformations classically seen in MPS I. Therefore, a skeletal X-ray is useful to improve the diagnostic rate in suspect cases [3,10,11,16] of MPS Ia [3].

The most significant radiological findings in MPS I patients are [1]:

Spine: thoracolumbar gibbus and/or anterior vertebral body breaking; kyphoscoliosis;

Long bones: short diaphysis, hypoplastic epiphyses, Madelung deformity;

Thorax: paddle-shaped ribs, short and thick clavicles;

Pelvis: hip dysplasia, coxa valga, rounded iliac wing, irregular dysplastic acetabuli;

Knee: genu valgum.

In MPS Ia, dysostosis multiplex could be less severe and slowly progressive than in severe forms and the radiologic findings are more difficult to recognize [3].

**Figure 1:**
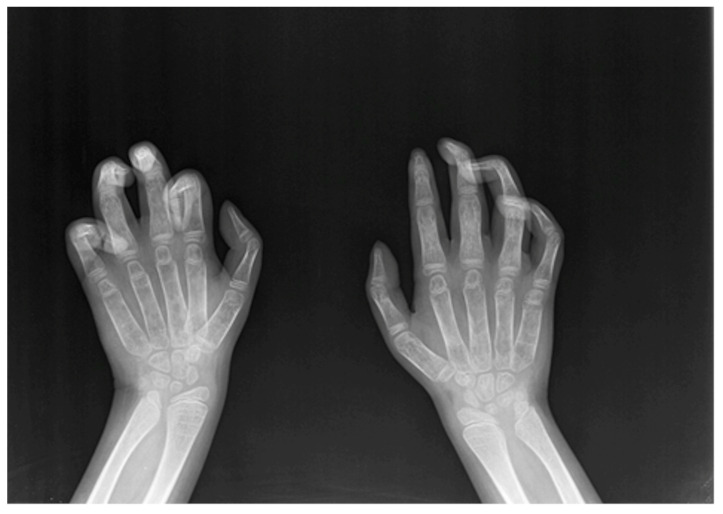
Claw hand and clinodactyly of the left II, III, IV, V finger and clinodactyly of the right III, IV, V finger.

As paediatric endocrinologists often prescribe a hand–wrist X-ray to assess bone age in children with impaired growth, MPS I-specific radiologic signs, such as bullet-shaped metacarpal bones (“bullet sign”), “claw hand”, clinodactyly (Figure 1), and “pseudo-Madelung” deformity of the distal radius should be promptly recognized by a skilled radiologist. Given the milder MPS Ia bony involvement, the above-mentioned findings may be attenuated and the clinical suspicion should be raised by slighter signs, such as isolated delayed ossification of the carpus, short metacarpal epiphyses, thickened wide and irregular metacarpals, and thickened phalanges [14].

## 4. The Algorithm

**Figure 2:**
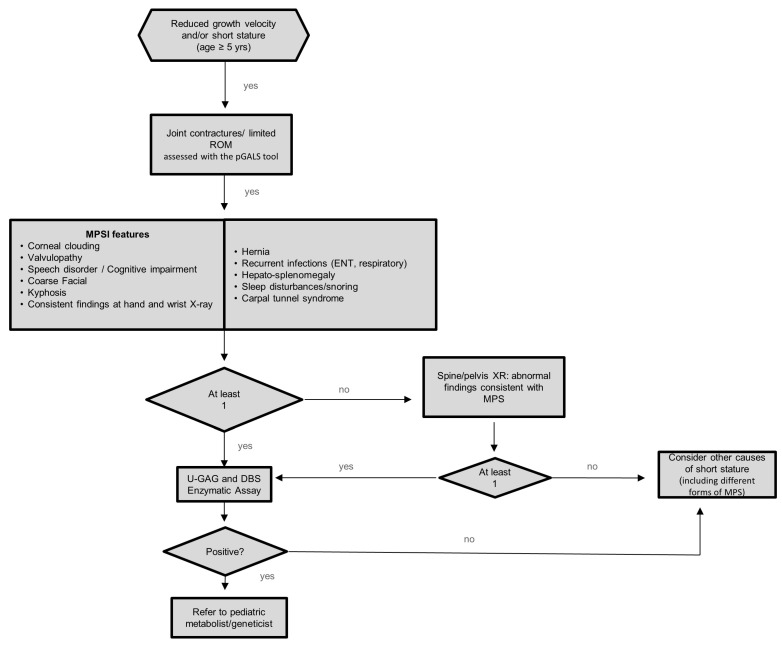
Algorithm for identification of MPS Ia in children with short stature and/or impaired growth. pGALS: pediatric Gait, Arms, Legs, and Spine. ROM: range of motion; DBS: dried blood spot; MPS: mucopolysaccharidosis; GAG: glycosaminoglycan; ENT: ear, nose, throat.

As demonstrated by the algorithm summarized in Figure 2, we recommend increasing the detection rate of MPS Ia in patients aged 5 years or older who are referred for either short stature and/or reduced growth velocity and/or height SDS remarkably below mid parental height SDS.

The finding of joint contractures and/or limited ROM should prompt a thorough clinical history collection and careful physical examination meant to recognize associated signs and symptoms consistent with a diagnosis of MPS I (Table 1).

In patients showing at least one additional feature among those listed in Table 1, urinary GAG and DBS for the enzymatic activity should be performed. In those without additional clinical criteria, skeletal radiological imaging, including hand and wrist X-ray, anteroposterior and lateral X-ray of the spine and pelvis should be obtained to detect typical but still clinically silent bone abnormalities suggestive of MPS I [11,17,18].

In case of consistent radiologic findings, the patients should undergo urinary GAG and DBS evaluation.

As shown in the algorithm, all patients who do not fulfil the criteria listed above should be fully investigated for other causes of short stature or growth delay. In case both urinary GAG and serum DBS provide a negative result, other metabolic storage disorders should be excluded.

## 5. Discussion

Short stature and impaired height velocity are common findings in MPS Ia and can become overt either during pre- or post-pubertal age. According to the data reported by Viskochil and colleagues, the estimated median height for untreated MPS Ia patients falls below the third centile by the age of 9 years [6].

Thus, it is overall likely that MPS Ia patients, still undiagnosed due to attenuated phenotypes, are referred to paediatric endocrinologists at a variable age with complaints of faltering growth.

Our algorithm emphasizes the importance of detecting impaired joint mobility as a pivotal feature of MPS Ia for the paediatric endocrinologist. In a series of patients with MPS Ia, the median age upon diagnosis was 9.8 years, but the median age upon first complaints of clinical signs and symptoms was 5.4 years [9]. Interestingly, 63% of patients diagnosed before the age of 5 years already showed clinical symptoms classically associated with MPS I, such as hernia or coarse facial features, whereas joint contracture was the most prevalent sign in patients diagnosed between 5 and 10 years. The median age of diagnosis of CTS was 13.1 years, while dysostosis multiplex and valvulopathy were identified after the age of 20. These results were confirmed in a large series of patients from the MPS I Registry [19].

For these reasons, we argued that the majority of patients not previously diagnosed with classic MPSI symptoms would probably undergo a paediatric endocrinology evaluation for short stature or blunted growth at an age above 5 years and probably show joint contractures and limited ROM.

So far, other flowcharts have been published to improve the early diagnosis of attenuated forms of MPS [8,10]. In detail, in an algorithm recently published by Guffon and colleagues [14], the Authors suggested that urinary GAG or enzyme activity were assessed only after radiological confirmation of dysostosis multiplex in suspected cases with impaired growth and joint contractures. Conversely, we regarded the finding of faltering growth plus joint contractures, as a strong indicator to assess urinary GAG and enzyme activity in patients with additional clinical features compatible with MPS Ia. Overall, we focused upon the pivotal role of clinical data, while the role of total body X-ray survey was considered as a screening tool in cases with unrecognized clinical features of MPS I. Hand and wrist X-ray is generally prescribed by paediatric endocrinologists to assess bone age in any patient with short stature and, for this reason, we included it among the first-level assessments and clinical features in the algorithm. In our opinion, spine and pelvis radiograms should be performed only in highly suspicious cases without radiological abnormalities in the hand and wrist. This approach could be time sparing and potentially reduces the amount of ionizing radiation. The present algorithm, born by a multidisciplinary team following a consensus-based methodology, is mainly addressed to paediatric endocrinologists. It could be more practical as compared to others and we suppose it may be helpful to diagnose a case with MPS Ia that comes to the attention of the paediatric endocrinologist for short stature or impaired growth velocity, without having previously identified the classic signs of MPS I, even if attenuated. Investigations specific for MPS Ia (DBS, urine GAG) follow targeted anamnestic data, a simple general and musculoskeletal evaluation by pGALS [15], and an X-ray of the hand and wrist, with no need for more extensive radiological imaging, as this is not always easily interpreted by non-MPS-committed radiologists [20].

In addition, the combined assessment of urinary GAG and DBS for enzyme activity assay, as proposed in our algorithm, is more sensitive and allows the detection of those MPS Ia patients who present with falsely low GAGs. Moreover, DBS would allow performing molecular analysis confirmation without recalling the patients with low enzyme activity. Furthermore, if L-iduronidase activity is within the normal range and the clinical suspicion is still high, other types of MPS should be excluded and the patient should be referred to the metabolic diseases specialist for more specific genetic tests.

## 6. Conclusions

Based on the experience of a multidisciplinary panel of experts, we hereby proposed a diagnostic algorithm in order to improve the awareness of paediatric endocrinologists towards attenuated MPS I and to prompt earlier diagnoses among patients evaluated for growth impairment as a presenting sign.

We believe that this algorithm will help to improve the awareness of paediatric endocrinologists towards attenuated MPS I.

We intend to disseminate the algorithm among paediatric endocrinologists in Italy to validate it prospectively in the future.

## Figures and Tables

**Table 1:** Most common signs and symptoms of severe and attenuated MPS at diagnosis.

	Severe MPS I (Hurler)	Intermediate MPS I (Hurler/Scheie)	Attenuated MPS I (Scheie)
Carpal tunnel syndrome	+	++	+++
Recurrent orthopedic or ENT surgeries	+	++	+++
Corneal clouding	+++	+++	+++
Cardiac valve disease and heart hypertrophy	+++	+++	+++
Hernias	++	++	++
Coarse face or facial dysmorphism	+++	+++	+
Spine kyphosis, gibbus	+++	++	+
Sleep disturbances/snoring	++	++	+
Recurrent ENT infections	+++	+++	+
Hepato-splenomegaly	+++	+++	+
Cognitive impairment/speech disorder	+++	+	-

Mucopolysaccharidosis Type I (MPS I) +++ frequency greater than 60%; ++ between 50 and 60%; + between 40 and 50%, ENT: ear, nose, throat.

## Data Availability

Not applicable.

## Abbreviations

CTS	carpal tunnel syndrome
DBS	dried blood spot
ERT	Enzyme Replacement Therapy
GAGs	glycosaminoglycans
MPS	Ia attenuated mucopolysaccharidosis type I
ROM	range of motion
ST	stenosing tenosynovitis
pGALS	paediatric Gait, Arms, Legs, and Spine
